# A new SPECT/CT reconstruction algorithm: reliability and accuracy in clinical routine for non-oncologic bone diseases

**DOI:** 10.1186/s13550-018-0367-7

**Published:** 2018-02-12

**Authors:** Olivier Delcroix, Philippe Robin, Maelenn Gouillou, Alexandra Le Duc-Pennec, Zarrin Alavi, Pierre-Yves Le Roux, Ronan Abgral, Pierre-Yves Salaun, David Bourhis, Solène Querellou

**Affiliations:** 1 Nuclear Medicine Department, CHRU Hospital Morvan, Brest, France; 2 CHRU Brest, Brest, France; 3 Service de Médecine Nucléaire, EA 3878 GETBO IFR 148, Brest, France; 4 Université de Bretagne Occidentale, Brest, France; 5 Brest Medical University Hospital—Inserm CIC 1412, Brest, France; 6 Nuclear Medicine Department, University Hospital, Boulevard Tanguy Prigent, 29200 Brest, France

**Keywords:** SPECT/CT, Bone diseases, Diagnostic accuracy, Scintigraphy, xSPECT Bone®, Reconstruction algorithm

## Abstract

**Background:**

xSPECT Bone® (xB) is a new reconstruction algorithm developed by Siemens® in bone hybrid imaging (SPECT/CT). A CT-based tissue segmentation is incorporated into SPECT reconstruction to provide SPECT images with bone anatomy appearance. The objectives of this study were to assess xB/CT reconstruction diagnostic reliability and accuracy in comparison with Flash 3D® (F3D)/CT in clinical routine. Two hundred thirteen consecutive patients referred to the Brest Nuclear Medicine Department for non-oncological bone diseases were evaluated retrospectively. Two hundred seven SPECT/CT were included. All SPECT/CT were independently interpreted by two nuclear medicine physicians (a junior and a senior expert) with xB/CT then with F3D/CT three months later. Inter-observer agreement (IOA) and diagnostic confidence were determined using McNemar test, and unweighted Kappa coefficient. The study objectives were then re-assessed for validation through > 18 months of clinical and paraclinical follow-up.

**Results:**

No statistically significant differences between IOA _xB_ and IOA F3D were found (*p* = 0.532). Agreement for xB after categorical classification of the diagnoses was high (*κ*_xB_ = 0.89 [95% CI 0.84 –0.93]) but without statistically significant difference F3D (*κ *_F3D_ = 0.90 [95% CI 0.86 – 0.94]). Thirty-one (14.9%) inter-reconstruction diagnostic discrepancies were observed of which 21 (10.1%) were classified as major. The follow-up confirmed the diagnosis of F3D in 10 cases, xB in 6 cases and was non-contributory in 5 cases.

**Conclusions:**

xB reconstruction algorithm was found reliable, providing high interobserver agreement and similar diagnostic confidence to F3D reconstruction in clinical routine.

## Background

**Fig. 1:**
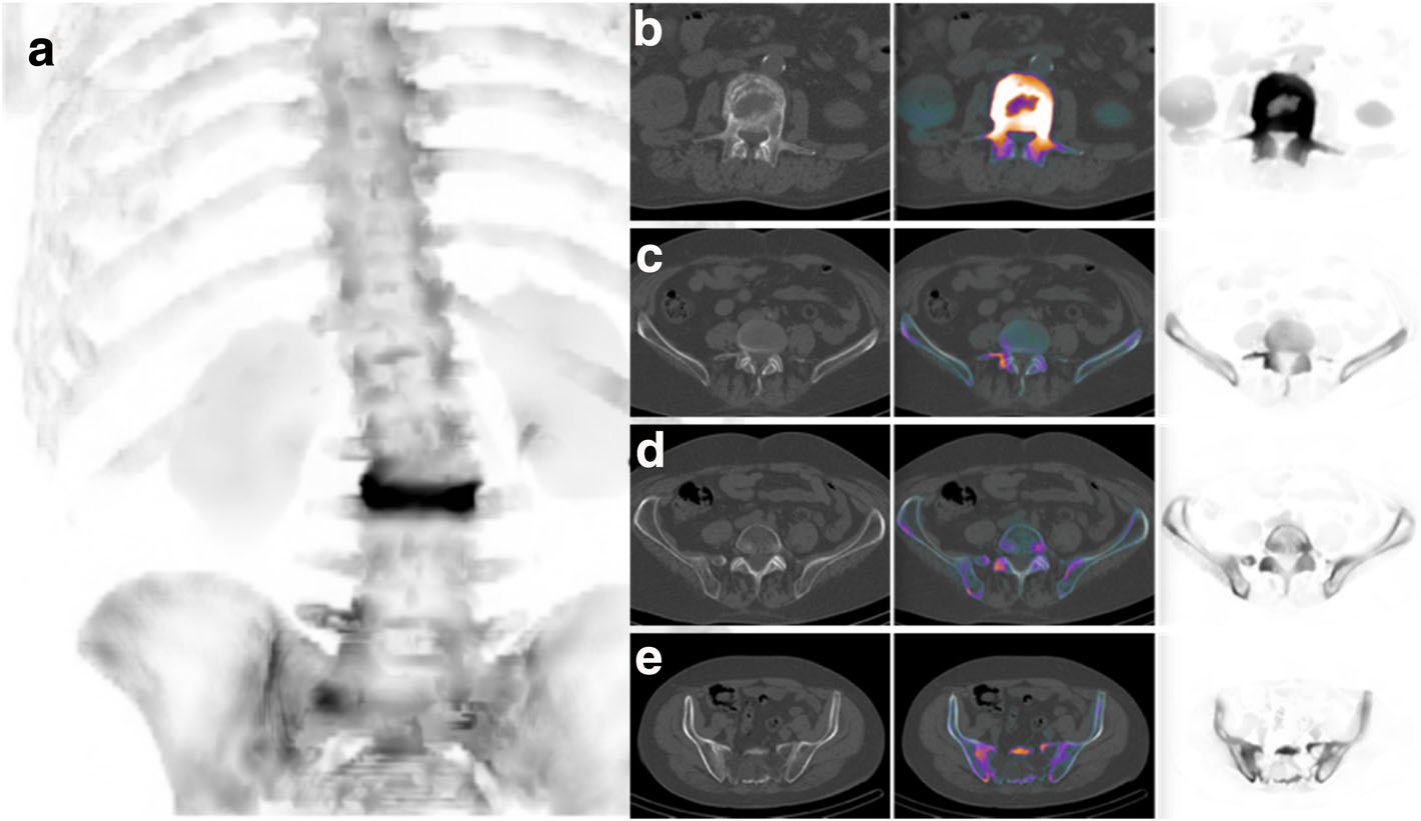
Example of an xSPECT Bone reconstruction providing SPECT images with bone anatomy appearance. The xSPECT Bone® maximum intensity projections (MIP) combines scintigraphic data with morphological data from the computed tomography. In this example, the scintigraphy was performed in order to explore an acute low back pain. The exclusive use of the xSPECT Bone® MIP (**a**) makes it possible to visualize on one image an uptake related to a fracture of a vertebral body responsible for spinal angulation, to identify the vertebra concerned (L3), to visualize an uptake localized on the fifth right transverse process extended to the zygapophysial joint, and to visualize an uptake of the last right zygapophysial joint and an uptake asymmetry of the sacroiliac joints. Transaxial image analysis confirms the fracture of the L3 vertebral body (**b**) and specifies its extension toward the pedicles. It also confirms the uptake of the fifth right transverse process (**c**), of the last right zygapophysial joint (**d**) and the uptake asymmetry of the sacroiliac joints (**e**)

xSPECT Bone® (xB) is a new iterative reconstruction algorithm developed by Siemens® for bone single photon emission computed tomography (SPECT). Unlike classic SPECT reconstructions, xB uses ordered subset conjugate gradient minimization algorithm (OSCGM). Its originality consists of constraining counts in computed tomography (CT) based on bone segmentation (Fig. 1) and providing a quantitative reconstruction [1, 2].

This innovation, like the progress of image acquisition and reconstruction, could convey a higher diagnostic confidence through an enhanced bone uptake location. Studies have reported early planar images with good sensitivity yet poor specificity. The latter was improved when using SPECT reconstructions with negative predictive value while maintaining an excellent sensitivity [3–5]. Moreover, the use of CT improved the specificity of SPECT [4], particularly concerning small lesions. Besides, physical limitations such as attenuation or Compton scattering have also benefited from corrections integrated directly into reconstruction algorithms, leading to less artifacts and shorter reconstruction time. Then, the “side-by-side” display of SPECT and CT images (SPECT + CT) was replaced by fused SPECT/CT images [6–10]. In this manner, Römer et al. were able to identify 90% of SPECT findings classified as indeterminate [11]. These authors also indicated that exact matching of functional and anatomic data may be necessary, especially for imaging of small anatomic structures.

That said, taking into account patient’s clinical data should also be regarded as a mainstay in enhancement of overall diagnostic confidence of scintigraphy.

In the end, in non-oncological context, the objective of both clinician and health care provider is to reduce additional imaging that could delay patient management, increase stress, and induce additional irradiation.

The objectives of this study were:

- First, to evaluate the reliability of xB/CT bone reconstruction in comparison with that of Flash 3D® (F3D)/CT.
- Second, to evaluate the diagnostic confidence of xB/CT compared with that of F3D/CT for non-oncological painful bone diseases according to the recommendations of good practice of the European Association of Nuclear Medicine [12].

## Methods

### Patients

A retrospective study was conducted on 213 non-oncological patients referred for a bone scintigraphy at the Nuclear Medicine Department of Brest University Hospital from March to September 2014. Seven patients were excluded (four due to a poor image fusion between SPECT and CT related to important movements, one for whom the SPECT/CT was not retrieved from PACS (picture archiving and communication system), another for whom the field of view of the CT was too small, and finally one who declined to participate in the study). All patients were given verbal information before the exams that their data could be used for future scientific research and gave their written consent.

The SPECT/CT of 206 patients was analyzed (70 male and 136 female) with 13 patients younger than 18 years old. Their mean age ± SD was 53.2 ± 18.8.

Two hundred seven SPECT/CT were included for 206 patients (2 SPECT/CT performed for the same patient). The anatomical areas explored are summarized in Table 1.

**Table 1:** Anatomical areas explored

SPECT/CT	207
Hip and pelvis	32
Elbow	1
Shoulder	8
Knee	20
Hand—wrist	25
Foot—ankle	101
Spine	11
Tibia	1
Chest	8

### Imaging acquisition

SPECT/CT data were acquired between 2 and 4 h after the intravenous injection of approximately 9 MBq/kg of ^99m^TcDPD (TECEOS®, CIS bio-international, 91112 Gif-Sur-Yvette, France) on a Symbia Intevo T6 dual-headed gamma camera (Siemens® SAS Medical Solutions, Munich, Germany) equipped with a low-energy high-resolution parallel-hole collimator. The energy window was set at 15%, centered on the photon energy peak of ^99m^Tc (140 keV).

The SPECT acquisition protocol was as follows: 60 frames per detector head, each with duration of 10 s, were acquired in step-and-shoot mode over 360° with non-circular orbit. Acquisition matrix was 256 × 256 to allow xB reconstruction.

The CT acquisition was performed immediately after the SPECT acquisition as follows: the image matrix size was 512 × 512, with a tube voltage of 110 kV for the extremities and 130 kV out of the extremities; automatic exposure control system (CARE Dose4D) with 90 quality reference mAs; a pitch of 1.05 for the extremities and 1.0 out of the extremities; a slice thickness of 5 mm for attenuation correction (AC), 1.25 mm for the extremities, and 3 mm out of the extremities; and a field of view of 30 cm for the extremities and 50 cm out of the extremities including the knees. FBP reconstructions were used with a B31s filter for AC, IRIS iterative reconstructions with i30s and i80s filters for analysis.

The SPECT/CT acquisition for the wrist and hand was performed on prone position.

### Reconstructions xB and F3D

The goal of iterative reconstruction is to find the best estimated slice that, when projected in multiple directions, is as close to acquired projections as possible. xSPECT is based on OSCGM algorithm, and xB is a variant of xSPECT® that considers that almost all the ^99m^Tc-DPD is localized in bones. First, CT is re-sampled to xSPECT® resolution (256^2^). CT data are then segmented in five increasing DPD uptake probability areas (air, adipose tissue, soft tissue, spongy bone, cortical bone). Those probabilities are incorporated in the xB algorithm, which constrains the reconstructed data in high uptake probability area, especially bones. To speed up the computation, ordered subsets can also be used [1].

The xB reconstruction was first performed with 36 iterations and 1 subset, a 256 × 256 matrix that leads to a 2.4-mm pixel and a 10-mm full width at the half maximum (FWHM) Gaussian post-filter. Then, an undersampling from 256^2^ to 128^2^ was performed on the projections in order to perform F3D reconstruction, with 8 iterations, 15 subsets, and a 12-mm FWHM Gaussian post-filter.

### Image analysis

Co-registered CT, SPECT, and SPECT/CT images were visualized with a commercially available 3D volume fusion tool (Syngo.via®, Siemens Healthcare). The 3D images were displayed as 2D orthogonal (axial, coronal, and sagittal, automatically generated by multiplanar reformatting (MPR) from the axial slices) and maximum intensity projections (MIP) on two screens through a custom display, allowing spatial synchronization through a triangulation pattern. The look-up table of the SPECT/CT images was “Warm Metal.”

### Interpretation and analysis of data

Retrospectively, all bone scans were independently interpreted by two nuclear medicine physicians (one senior physician (experience of 15 years) and one junior physician (experience of 3 years)) with the xB/CT reconstruction and then 3 months later in order to obtain a blind interpretation of the first one with F3D/CT reconstruction. Choosing a junior and a senior physician would allow assessment of the diagnostic confidence reliability and accuracy in bone reconstruction, whatever the reader’s experiences. Each bone scintigraphy was interpreted by simultaneous analysis of the SPECT, CT, and SPECT/CT reconstructions. The interpretation was made with the knowledge of the clinical context, i.e., our clinical routine.

The diagnoses were classified into five categories: 1—normal scintigraphy, 2—arthritis, 3—periarticular disease, 4—fracture or tumor pathology, and 5—complex regional pain syndrome. The diagnoses are summarized in Table 2.

**Table 2:** Categorical classification of the diagnosis

1	Normal scintigraphy	No pathological uptake CT anomaly without uptake on the SPECT Non-pathological bone remodeling, after surgery for instance
2	Articular disease	Arthrosis Os trigonum syndrome Sacroiliitis Infectious arthritis Joint manifestation of alkaptonuria Prosthesis failure Stress shielding Bone–prosthesis conflict
3	Periarticular disease	Heel spur Rheumatic disease Plantar fasciitis Para-osteo-arthropathy
4	Fracture or tumor pathology	Fracture Osteochondral lesion of the talar dom Micro fracture Pseudarthrosis Fibrous dysplasia of bone Osteitis Osteoid osteoma Osteonecrosis
5	Complex regional pain syndrome	Complex regional pain syndrome

First, the interpretation discrepancies between the two physicians were identified within xB and within F3D. In case of diagnostic discrepancy between the two physicians, the diagnosis was made after consensus. In a second step, the diagnostic differences between xB and F3D reconstructions after harmonization within each reconstruction were identified. They were classified as major (if the diagnosis and the treatment were different) or minor (if they were irrelevant and did not lead to any therapeutic modification).

### Clinical and paraclinical follow-up

For all included patients, the follow-up was carried out either by consulting the medical file or by calling the referring physician and/or the patient directly. Due to the retrospective nature of our study, the referring physician had already received the F3D reconstruction report. The clinical and paraclinical follow-up was carried out over 18 months after scintigraphy of the last patient. Of the 206 patients, 204 were followed up. Two patients were lost to follow-up. Patients’ additional data are summarized in Table 3.

**Table 3:** Clinical and paraclinical follow-up

Plain radiograph	104
Magnetic resonance imaging	45
Computed tomography (CT)	34
CT arthrography	9
Ultrasound	25
Electromyography	2
New bone SPECT/CT	15
Bacteriological analysis	3
Clinical follow-up alone	47

The diagnostic differences (major or minor) between xB and F3D reconstructions were compared with the clinical and paraclinical follow-up, considered as the reference standard in our study.

### Statistical analysis

The comparison of the IOA by reconstruction was performed according to two distinct statistical methods: raw diagnoses were compared with a McNemar test and diagnostic categories were compared with an unweighted kappa coefficient (according to the five categories mentioned above).

The retrospective nature of the study did not allow us to have a reference standard independent from the index test. Indeed, for situations in which a difference in diagnosis was observed between xB and F3D reconstructions, a simple descriptive comparison with the follow-up was performed.

## Results

### Inter-observer agreement (IOA) and inter-observer discrepancy (IOD)

Among the 207 SPECT/CT interpreted with xB then with F3D, 23 IOD were observed within F3D without IOD for these same 23 cases with xB, thus representing 11.1% of IOD in the F3D arm. Similarly, 18 IOD were observed within xB without IOD for these same 18 cases with F3D, representing 8.7% of IOD in the xB group. For the remaining 166 examinations, no IOD was found in both xB and F3D (Table 4).

**Table 4:** Inter-observer agreement between xSPECT Bone® and Flash 3D® reconstruction algorithms

xSPECT Bone®	Flash 3D®	
	Concordant	Discrepancy
Concordant	166	23
Discrepancy	18	0

A McNemar test showed no statistically significant difference between IOA _xB_ and IOA _F3D_ (*p* = 0.532).

Moreover, the unweighted kappa coefficient calculated after categorical classification of the diagnoses was high but did not demonstrate a statistically significant difference between F3D and xB: kappa _F3D_ = 0.90 [95% CI 0.86–0.94] and kappa _xB_ = 0.89 [95% CI 0.84–0.93]. The contingency table of the diagnosis is presented in Table 5, according to the two physicians after categorical classification of the diagnosis.

**Table 5:** The contingency table of the diagnosis according to the two physicians after categorical classification of the diagnosis (262 lesions were observed for 207 SPECT/CT)

Physician 2	Physician 1									
	Flash 3D®					xSPECT Bone®				
Categorical diagnosis	1	2	3	4	5	1	2	3	4	5
1	62	3	0	2	1	63	7	1	3	1
2	0	79	0	1	2	0	74	0	2	0
3	1	0	13	0	0	0	0	13	0	0
4	4	5	1	57	0	2	4	1	59	1
5	0	0	0	0	31	0	0	0	0	31

### Inter-reconstruction diagnostic discrepancy (IRDD)

Thirty-one (14.9%) IRDD were observed out of 207 SPECT/CT, with raw diagnosis or categorical diagnosis. Twenty-one (10.1%) IRDD were classified as major and 10 (4.8%) IRDD as minor.

Among the 21 major IRDD, the follow-up confirmed the diagnosis of F3D in 10 cases and xB in 6 cases and was non-contributory in 5 cases. Of the 16 cases for which follow-up was informative, there were 5 false negatives for F3D and 4 false negatives for xB, 4 false positives for xB but none for F3D and 3 localization errors, 2 for xB and one for F3D. IRDD are described in Table 6.

**Table 6:** Inter-reconstruction diagnostic discrepancy

	Symptoms	xSPECT Bone® abnormalities	Flash 3D® abnormalities	Diagnosis*	Error
	Discrepancy between Flash 3D and diagnosis*				
1	Hip pain	Right hip uptake	No pathological uptake	Right hip arthrosis	F3D-false negative
2	Right ankle pain	Right os trigonum syndrome	Right talus contusion	Right os trigonum syndrome	F3D-location
3	Left knee joint pain	Uptake of fracture sequelae of patella	No pathological uptake	Knee arthritis	F3D-false negative
4	Left gluteal region pain	Left sacroiliac joint uptake	No pathological uptake	Sacroiliac arthritis	F3D-false negative
5	Left knee joint pain, intercondylar eminence fracture several months ago	Intercondylar eminence uptake	No pathological uptake	Intercondylar eminence pseudarthrosis	F3D-false negative
6	Lumbar pain	Zygapophyseal arthritis	No pathological uptake	Zygapophyseal arthritis	F3D-false negative
	Discrepancy between xSPECT Bone® and diagnosis*				
7	Left ankle pain	Tarsometatarsal arthritis	No pathological uptake	Fibromyalgia	xB-false positive
8	Chronic left ankle pain	No pathological uptake	Calcaneus fracture	Fracture	xB-false negative
9	Right hip pain, prosthesis	No pathological uptake	Hip uptake	Prosthesis failure	xB-false negative
10	First tarsometatarsal pain	No pathological uptake	Tarsometatarsal uptake	Tarsometatarsal arthritis	xB-false negative
11	Feet pain	Micro fracture of the head of the 2nd metatarsal	2nd metatarso-phalangeal joint uptake	Arthritis	xB-location
12	Feet pain	Micro fracture of cuboid bone	No pathological uptake	Spontaneous disappearance of pain	xB-false positive
13	Left scapula pain	Supraspinatus tendinopathy	No pathological uptake	Spontaneous disappearance of pain	xB-false positive
14	Left ankle pain	No pathological uptake	Plantar fasciitis	Plantar fasciitis	xB-false negative
15	Right wrist pain	Lunate bone fracture	Lunate–capitate bone conflict	Pseudarthrosis	xB-location
16	Distal left thumb pain	Osteitis of the last phalange	No pathological uptake	Conversion disorder	xB-false positive
	Non-informative clinical and paraclinical follow-up				
17	Left foot pain	Sesamoide bone contusion	Tarsometatarsal arthritis		
18	Left ankle pain	Talocrural arthritis with malleolus fracture	Talocrural arthritis without malleolus fracture		
19	Left ankle pain	Tibia fracture	Talocrural arthritis		
20	Right tibia pain	Talus fracture	No pathological uptake		
21	Right first metatarsal bone pain	Sesamoide–metatarsal bone conflict	Fracture of the head of the first metatarsal bone		

*F3D* Flash 3D®, *xB* xSPECT Bone®

*Diagnosis was done thanks to clinical and paraclinical follow-up

### IOD–IRDD relations

Forty-one (19.8%) IOD were observed for the 207 SPECT/CT. For the 31 IRDD, 13 IOD (41.9%) were observed. Seven IOD (33.3%) were observed for the 21 major IRDD, and 6 IOD (60%) were observed for the 10 minor IRDD.

### Analysis of scintigraphy with bone prosthesis

Twenty-four bone scans concerned an exploration of pain involving joints with prosthetic replacement. Three IOD (12.5%) were identified (one for xB and two for F3D). Only one IRDD was identified. Follow-up concluded to a false negative of xB. No false positive was identified with xB. It should be noted that for four scans with concordant findings xB and F3D, follow-up was contradictory (three false negatives and one false positive results).

## Discussion

The Siemens® xB tomographic image reconstruction is a new way of bone image reconstruction theoretically being suggested to provide better bone contrast, thus high-quality images compared with conventional reconstructions. This hypothesis should be assessed for confirmation. To our knowledge, this study is the first to evaluate the diagnostic reliability and accuracy of this novel reconstruction in routine clinical practice. The study includes a large number of patients and their follow-up and concludes to a high inter-observer agreement and a similar diagnostic confidence as compared with F3D.

A high kappa index for xB (0.89) [95% CI 0.84–0.93] showed a very strong IOA, highlighting the reliability of interpretation, between junior and senior expert readers.

The kappa index obtained according to F3D reconstructions was also high (0.90) with a confidence interval [95% CI 0.86–0.94] without statistically significant differences in inter-observer agreement. The same conclusions were obtained with the McNemar test (*p* = 0.532). We thus observed equivalent diagnostic confidence between xB and F3D reconstructions.

Thirty-one IRDD (14.9%) were observed among the 207 SPECT/CT. Of the 31 IRDD, 21 were classified as major (10.1%). A diagnosis was made according to the follow-up in most IRDD cases (16/21). With a better spatial resolution to observe smaller SPECT abnormalities and a better bone to soft tissue contrast, xB may theoretically allow increased detection and better visualization of weakly evolving or small abnormalities that could go unnoticed with F3D. However, according to our clinical experience, detecting smaller or weakly evolving abnormalities did not have a major clinical relevance and did not lead to a therapeutic modification.

Indeed, the higher the IRDD proportion, the higher is the IOD percentage (i.e., high IRDD 41.9% vs. low IRDD 19.8%): borderline bone scan abnormalities were most likely interpreted subjectively (i.e., between physicians with different experience) and therefore more likely to induce IOD, with less clinical relevance.

**Fig. 2:**
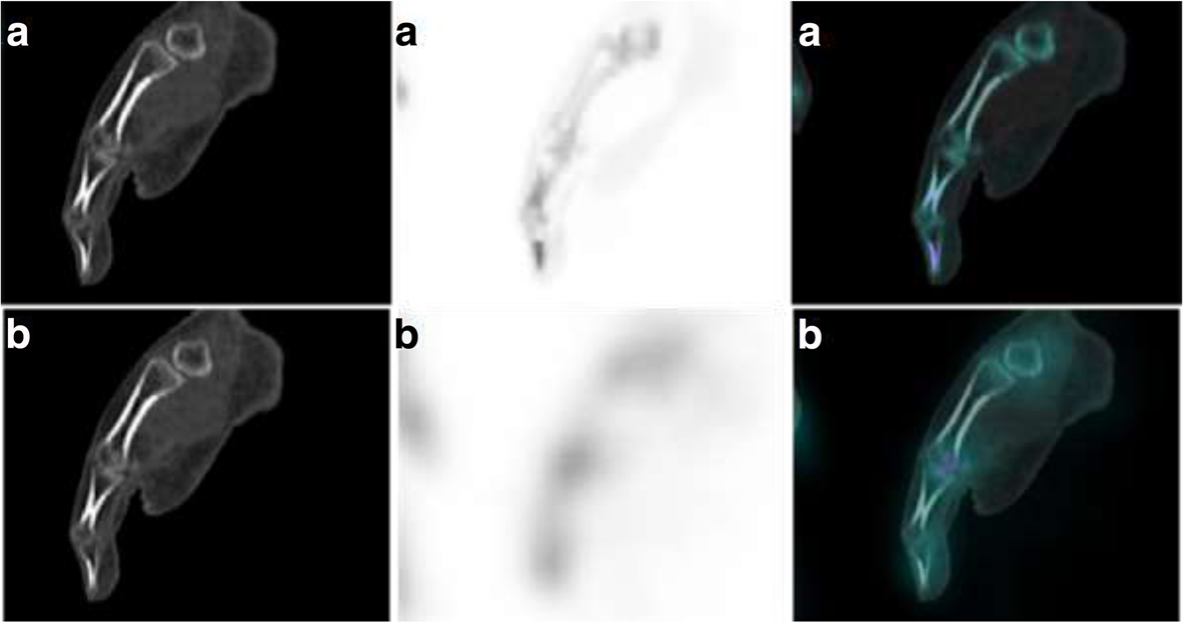
Example of false positive of xSPECT Bone®. This SPECT/CT was performed in order to explore a focal distal thumb pain persisting after a traumatism occurred several months ago. A moderate uptake involving only the last phalange of the thumb, higher than the other phalangeal uptakes, is observed on the xB image (**a**), matched with the focal pain and suggesting an osteitis. However, there is no pathological uptake on the F3D image (**b**). The MRI performed after SPECT/CT was normal and ruled out the osteitis. A conversion disorder was diagnosed

**Fig. 3:**
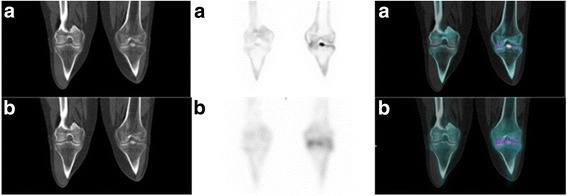
Example of false negative of Flash 3D ®. This SPECT/CT was performed in order to explore a left knee joint pain persisting after an intercondylar eminence fracture occurred several months ago. A focal intense uptake of the left intercondylar eminence is observed on the xB image (**a**) whereas a diffuse uptake of the tibiofemoral joint is observed on the F3D image (**b**). MRI realized after SPECT/CT confirmed the intercondylar eminence pseudarthrosis evoked on xB image

**Fig. 4:**
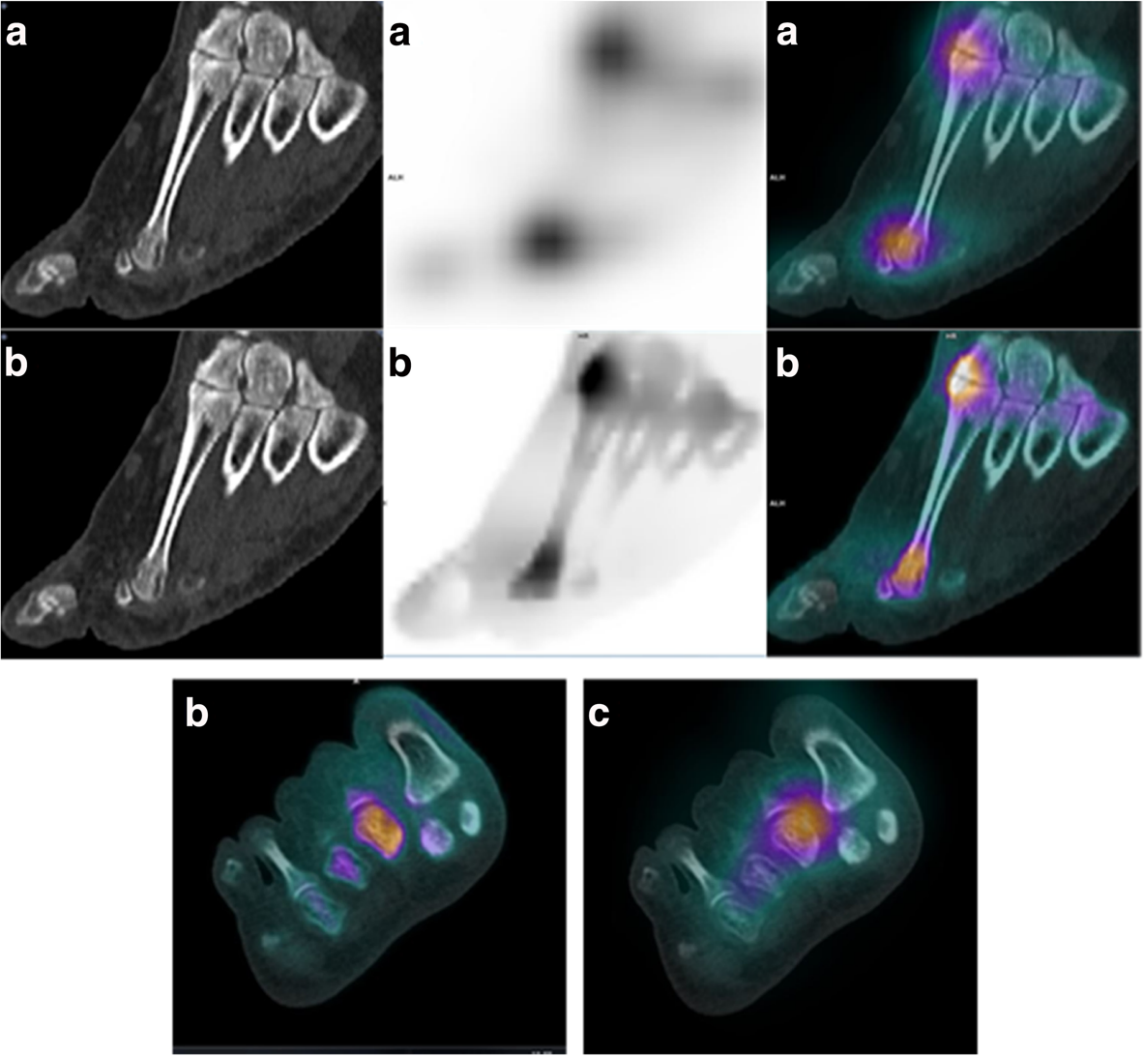
Location error of xSPECT Bone® related to the movements between SPECT and CT acquisition. This SPECT/CT was performed in order to explore a foot pain with suspicion of complex regional pain syndrome. An uptake of the second metatarso-phalangeal joint is observed on the F3D image (**a**) whereas an uptake of the head of the second metatarsal is observed on the xB image (**b**). The absence of traumatic context and the evolution with painful flares for 2 years suggests an osteoarthritic origin, confirming the hypothesis evoked by F3D/CT. Moreover, we can observe on the axial slice F3D/CT (**c**) a spatial shift between F3D acquisition and CT acquisition related to the movements of the patient, causing a bad reconstruction and a localization error of xB

**Fig. 5:**
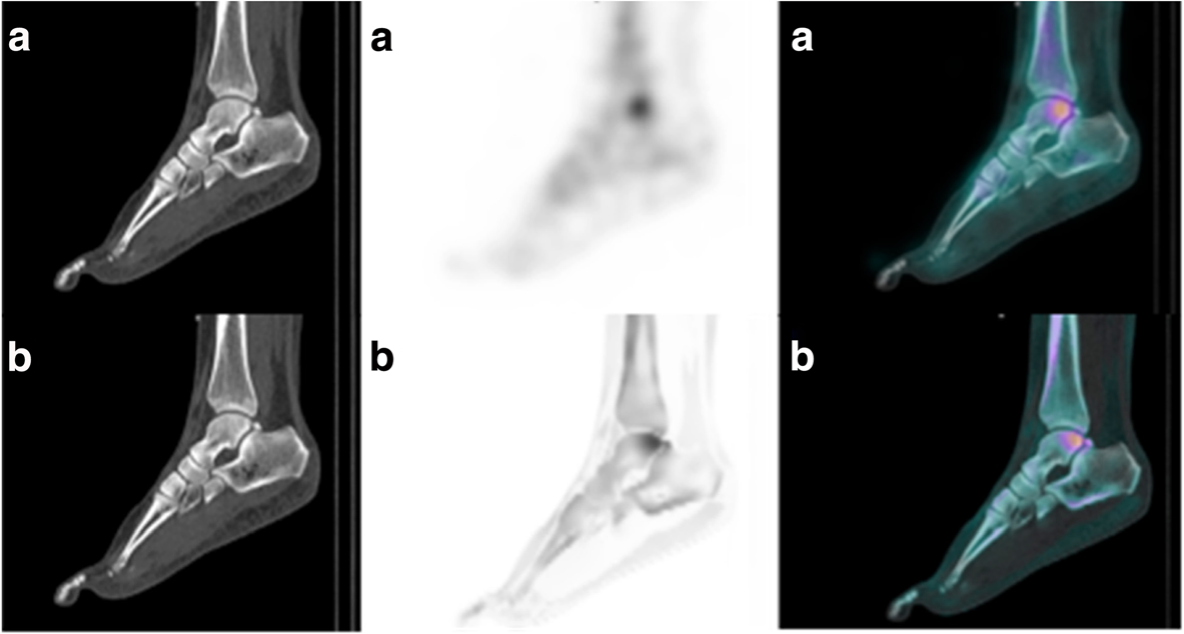
Example of a better location of a pathological uptake with xSPECT Bone® reconstruction. This SPECT/CT was performed in order to explore a chronic right ankle pain which appeared 1 year ago without traumatism. An uptake of the talus is observed on the F3D image (**a**) whereas an uptake of the talus and of a trigonum bone is observed on the xB image (**b**). MRI realized after SPECT/CT did not show contusion of the talus but confirmed the right os trigonum syndrome observed on xB image

Of the 16 IRDD, 10 diagnoses done by F3D vs. 6 by xB were confirmed through follow-up. This difference was explained in particular by a higher number of false positives for xB, 4 against none for F3D (Fig. 2). However, the number of false negatives was almost equivalent, 5 for F3D and 4 for xB (Fig. 3). Finally, three radiopharmaceutical uptake errors were observed: two in xB and one in F3D. One radiopharmaceutical uptake error in xB was due to patient movements between the SPECT and the CT acquisitions (Fig. 4). These were not detectable on xB/CT fused images alone but were detectable on F3D/CT images. Aberrant xB images due to location uptake errors are easy to identify. However, when the movements are minimal, they can be undetectable and lead to diagnostic error. This suggests the necessity to systematically take a look at the F3D/CT slices in order to control the accurate registration of SPECT and CT slices in xB reconstruction. Nevertheless, the good spatial resolution of xB can ease the reading and thus change the diagnosis. This is illustrated in Fig. 5: a joint disorder was diagnosed between talus and trigonum bones using xB (and confirmed by follow-up) and as a talus contusion using F3D.

It should also be noted that the study was not carried out by comparing only the SPECT reconstructions but rather by comparing the registered images SPECT/CT with knowledge of the clinical context. The use of CT slices and the knowledge of pain mechanism may have an impact on the diagnostic confidence of the scintigraphy. Thereby, Vija et al. [13] demonstrated significantly higher accuracy of xB used without CT slices compared to F3D. However, the difference was no longer statistically significant between the two reconstructions when fused with the CT slices.

Finally, the striking innovation of the xB reconstruction is the esthetic aspect of SPECT images, which could ease visualization and interpretation of anomalies on MIP images. This combined with clinical and paraclinical findings may enhance patient management and treatment.

Thus, the ease of interpretation provided by xB could bring an added value to the scintigraphic examination given the resultant high-quality images. Most clinicians pay close attention to the images and this often without reading the acquisition report [14].

Given the technological advancement in bone scintigraphy, clinicians and health care provider’s objectives are to highlight diagnostic confidence thus limiting the use of additional imagery.

All together, we believe that prospective studies are warranted to reach more conclusive results in regard with xB reconstruction reliability and accuracy in bone imaging. This further step can help at reaching robust clinical evidence as well as diagnostic consensus.

Similarly, to repeat this study in a multicentric way would limit the interpretation bias observed in our study. Our diagnostic decisions were not independent given that the junior physician was trained by the senior physician from our nuclear medicine department. Nevertheless, the inter-observer agreement scores are comparable to those observed in the literature (0.87–0.97) [8, 15–18].

## Conclusions

Our study demonstrated that xB reconstruction algorithm was a reliable tool in diagnosis of non-oncological bone diseases, providing high inter-observer agreement and similar diagnostic confidence compared with F3D. Moreover, it may improve SPECT/CT images quality thanks to a striking esthetic aspect. Moreover, the proper registration between SPECT and CT slices needs to be checked systematically in F3D images.
